# Characterization of nanoparticle mediated laser transfection by femtosecond laser pulses for applications in molecular medicine

**DOI:** 10.1186/s12951-014-0057-1

**Published:** 2015-02-03

**Authors:** Markus Schomaker, Dag Heinemann, Stefan Kalies, Saskia Willenbrock, Siegfried Wagner, Ingo Nolte, Tammo Ripken, Hugo Murua Escobar, Heiko Meyer, Alexander Heisterkamp

**Affiliations:** Department of Biomedical Optics, Laser Zentrum Hannover, Hollerithallee 8, 30419 Hannover, Germany; Small Animal Clinic, University of Veterinary Medicine Hannover, Bünteweg 9, 30559 Hannover, Germany; Department of Hematology, Oncology, and Palliative Medicine, University of Rostock, Ernst- Heydemann-Str. 6, 18057 Rostock, Germany; Department of Cardiothoracic Transplantation and Vascular Surgery, Hannover Medical School, Carl-Neuberg-Str. 1, 30625 Hannover, Germany; Institut für Quantenoptik Leibniz Universität Hannover Welfengarten 1, 30167 Hannover, Germany

**Keywords:** Laser transfection, Plasmonics, Nanoparticles, Permeabilization mechanisms, siRNA, Gene delivery

## Abstract

**Background:**

In molecular medicine, the manipulation of cells is prerequisite to evaluate genes as therapeutic targets or to transfect cells to develop cell therapeutic strategies. To achieve these purposes it is essential that given transfection techniques are capable of handling high cell numbers in reasonable time spans. To fulfill this demand, an alternative nanoparticle mediated laser transfection method is presented herein. The fs-laser excitation of cell-adhered gold nanoparticles evokes localized membrane permeabilization and enables an inflow of extracellular molecules into cells.

**Results:**

The parameters for an efficient and gentle cell manipulation are evaluated in detail. Efficiencies of 90% with a cell viability of 93% were achieved for siRNA transfection. The proof for a molecular medical approach is demonstrated by highly efficient knock down of the oncogene HMGA2 in a rapidly proliferating prostate carcinoma *in vitro* model using siRNA. Additionally, investigations concerning the initial perforation mechanism are conducted. Next to theoretical simulations, the laser induced effects are experimentally investigated by spectrometric and microscopic analysis. The results indicate that near field effects are the initial mechanism of membrane permeabilization.

**Conclusion:**

This methodical approach combined with an automated setup, allows a high throughput targeting of several 100,000 cells within seconds, providing an excellent tool for *in vitro* applications in molecular medicine. NIR fs lasers are characterized by specific advantages when compared to lasers employing longer (ps/ns) pulses in the visible regime. The NIR fs pulses generate low thermal impact while allowing high penetration depths into tissue. Therefore fs lasers could be used for prospective *in vivo* applications.

## Background

The direct modulation of gene expression is essential to establish therapeutic approaches in molecular medicine. Additionally to the development of therapies on the molecular level, the evaluation of target genes as therapeutic agents by combining the technology of RNAi and high throughput screenings is of major interest [1-3].

A major challenge in molecular medicine is the efficient, non-toxic and cell type independent transfection of cells in high throughput. In general a very effective manipulation strategy to achieve this is the transduction of cells via viral vectors. However, despite of the high efficiency this method bears high biological risk as integrational mutagenesis [4]. Alternative existing non-viral transfection methods show specific advantages and disadvantages. Transfection with lipid based reagents is often applied in high throughput assays but this method is cell type dependent and occasionally inefficient, especially for primary- and stem cell transfection [5]. Due to the difficulties in transfection of these cells, the commonly employed manipulation methods are either electroporation or nucleofection [6,7]. Unfortunately, these methods affect cell viability which is crucial when handling sensitive cells. Consequently in this manipulation it is essential to achieve a balance between transfection efficiency and methodical toxicity. Electroporation and nucleofection can also be utilized for high throughput assays, but these physical techniques remain usually limited to well plates with low well numbers being additionally cost ineffective [8].

In order to address these challenges methodicaly, a variety of optical transfection techniques have been developed based on pulsed as well as continuously emitting lasers [9-13]. None of these techniques fulfills the requirements of an efficient and low-toxic transfection method combined with high throughput. Accordingly, there is no laser based technique currently established, that allows routinely laboratory or clinical use. A promising tool for molecular medical applications is the nanoparticle mediated laser transfection using a microchip laser emitting ps laser pulses with a resonant wavelength of 532 nm [14,15]. Herein, gold nanoparticle (AuNP) labeled cells are irradiated with a weakly focused laser beam. This method allows targeting many cells simultaneously, ensuring high throughput while maintaining a high spatial selectivity. Additionally, this physical method using resonant laser pulses is very promising for the manipulation of a variety of cell types.

By applying off-resonant fs laser pulses, the transfection of hematopoietic stem cells (CD34+) can be achieved [16]. Here, the excitation of the membrane adhered AuNP with the incident laser light leads to plasmon resonances which increase the absorption and scattering cross section of the AuNP by several orders of magnitude. When the AuNP is irradiated at a resonant wavelength, the laser energy is absorbed leading predominantly to thermal effects and changes in the particles morphology [15,17]. Using near infrared (NIR) femtosecond (fs) laser systems, off-resonant AuNP excitation can be achieved [18]. At this wavelength the absorption and therefore the thermal impact is reduced and the incident light is scattered into the near field of the particle. Due to this “nanolens” effect, an enhancement of the electric field in the near field takes place [19]. If the AuNP is adhered to the cell membrane, the field enhancement can initiate a spatially confined membrane permeabilization [18]. In proof of principle experiments we could show the possibility to perforate the cell membrane using off resonant 800 nm fs laser pulses to deliver fluorescent labeled small interfering RNA (siRNA) and plasmid DNA (pDNA) into cells [20,21]. In another fs based study, a DNA-transfection rate of 23% using a melanoma cell line was stated and plasma induced nano-cavitation is supposed as the membrane permeabilization effect [22]. The advantage of NIR wavelengths located in the “diagnostic window” regime of the electromagnetic spectrum results in higher penetration depths into biological tissue which might allow *in vivo* applications [23]. Furthermore, the low absorption cross section in the NIR reduces the risk of thermal induced AuNP fragmentation.

**Figure 1:**
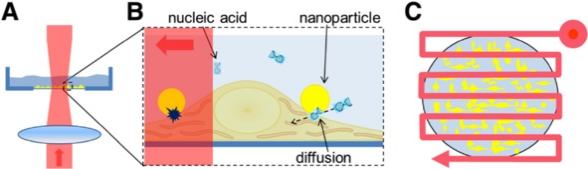
**Principle of AuNP mediated laser cell membrane permeabilization.** Spherical AuNP were incubated with the cells to allow sedimentation of the particles onto the cell membrane. Prepared samples were placed on an automatized stage to move selected wells of a well plate into the laser focus. Selected wells were completely irradiated by a raster shaped pattern with an inter line distance of 55 μm (1/3 of the laser diameter). **A)** Side view: the laser beam is weakly focused on the dish bottom where the AuNP labeled cells are located. **B)** Sketch of manipulation principle: AuNP are in contact with the cell membrane and irradiated by fs-laser pulses (left side). The interaction of the laser pulses with membrane adhered AuNP induces plasmon mediated effects which result in a transient enhanced permeability of the cell membrane. Through this permeabilization, extracellular molecules can cross the cell membrane and diffuse into the cytoplasm (right side). **C)** By applying a meander shaped scanning pattern, a high number of cells can be treated.

Within this work, microscopic analyses were performed to visualize the nanoparticle-cell membrane interaction, such that the co-incubation time for membrane permeabilization and the fundamental binding mechanism could be evaluated. To achieve an efficient uptake of extracellular molecules at high cell viabilities, a detailed parameter evaluation for a transient cell membrane permeabilization was performed. Different radiant exposures, scanning velocities of the laser spot, particle concentrations and particle sizes were applied to determine optimized permeabilization parameters. Additionally, the cell viability on a time scale up to 72 h after laser exposure and AuNP incubation was evaluated. The optimized parameters were used to evaluate the siRNA transfection efficiency, cell viability and functional oncogene knockdown in a cancer cell line. Due to the scanning method (Figure 1) and the automated setup, a high throughput is achieved and thus it is possible to handle all kinds of well plates within several minutes. Additionally to the manipulation experiments, the effects involved in the permeabilization process are investigated by temperature and near field simulations and a particle fragmentation study to further analyze the excitation of AuNP and the perforation mechanisms. The results indicate that both, near field and heating effects contribute to the mechanism of nanoparticle mediated membrane permeabilization in the fs regime.

## Results

### Interaction of cells with gold nanoparticles

**Figure 2:**
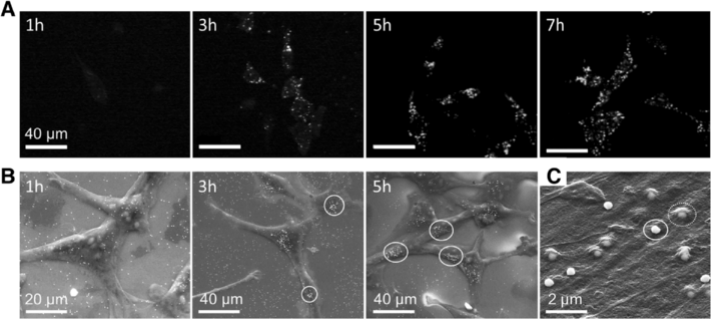
**Nanoparticle - cell interaction. A)** Time lapse multiphoton microscopy of granulosa cells with 150 nm particles after 1 h, 3 h, 5 h and 7 h of co-incubation. **B)** ESEM and **C)** SEM images of ZMTH3 cells after different incubation times with 200 nm gold particles. **B)** ESEM images: After 1 h a loose dispersion of particles is visible. After 3 h the AuNP started to aggregate (ellipse). The formation of particle clusters at the membrane can be observed after 5 h. **C)** SEM image in a higher magnification: After an incubation time of 3 h, particles are either on the cell membrane (solid ellipse) or covered by the membrane (dashed ellipse) [24].

Time lapse multiphoton microscopy was employed to monitor the incubation process. As shown in Figure 2A, bright spots, identified as the luminescence of the AuNP, are visible at the cell membrane after 3 h of incubation. Images which were taken at shorter incubation times show no spots or marginal changes in the background brightness. Increasing the incubation time from 3 to 5 h resulted slightly brighter luminescence. Within 5 to 7 h of co-incubation, the number and brightness of the AuNP signal saturated. The AuNP luminescence was still visible after washing, indicating that the particles remained adhered to the cell membrane.

Scanning electron microscopy (SEM) and environmental scanning electron microscopy (ESEM) provided detailed information about the attachment and distribution of the AuNP at the cell membrane after co-incubation and several washing steps (Figure 2B, C). The results show a loose dispersion of AuNP after 1 h of incubation. The particles were located at the culture dish bottom and on the cell membrane. By increasing the incubation time to more than 3 hours, the particles started to aggregate at the cell membrane. After an incubation time of 5 h, no further increase could be observed. Depending on the location of the particles, some of the particles appeared brighter than others. At higher magnifications, as visible in Figure 2C, some particles were located on the cell membrane (solid ellipse Figure 2C) and some were started to be endocytosed (dashed ellipse Figure 2C), which is demonstrated by the cell membrane covered particles. Based on this we defined an incubation time of 3 h for our gold nanoparticle mediated laser transfection. Within this time a sufficient number of particles adhere to the cell membrane to induce membrane permeabilization. The number of particles at the cell membrane was counted using ESEM images of ZMTH3 cells taken after 3 h of incubation. An incubation concentration of 11 μg/ml was applied which represents the optimal concentration for cell manipulation. On average 164 ± 50 particles at the membrane of a single cell were counted.

### Evaluation of efficient and gentle cell manipulation parameters

To evaluate the optimal process parameters for an efficient and gentle cell manipulation, the cells were treated with different parameters in the presence of 10 kDa FITC labeled dextran and the corresponding fluorescence level was determined. As an indicator for viability, the respective metabolic activities of the manipulated cells were measured after laser treatment using an fluorescence based assay (Qblue). An efficiency ratio of the used parameters was evaluated as the normalized ratio of FITC fluorescent level and viability. The purpose was to optimize the parameters for later transfection experiments and to get an overview of the influence of the different parameters. It was not intended to determine absolute transfection efficiencies.

**Figure 3:**
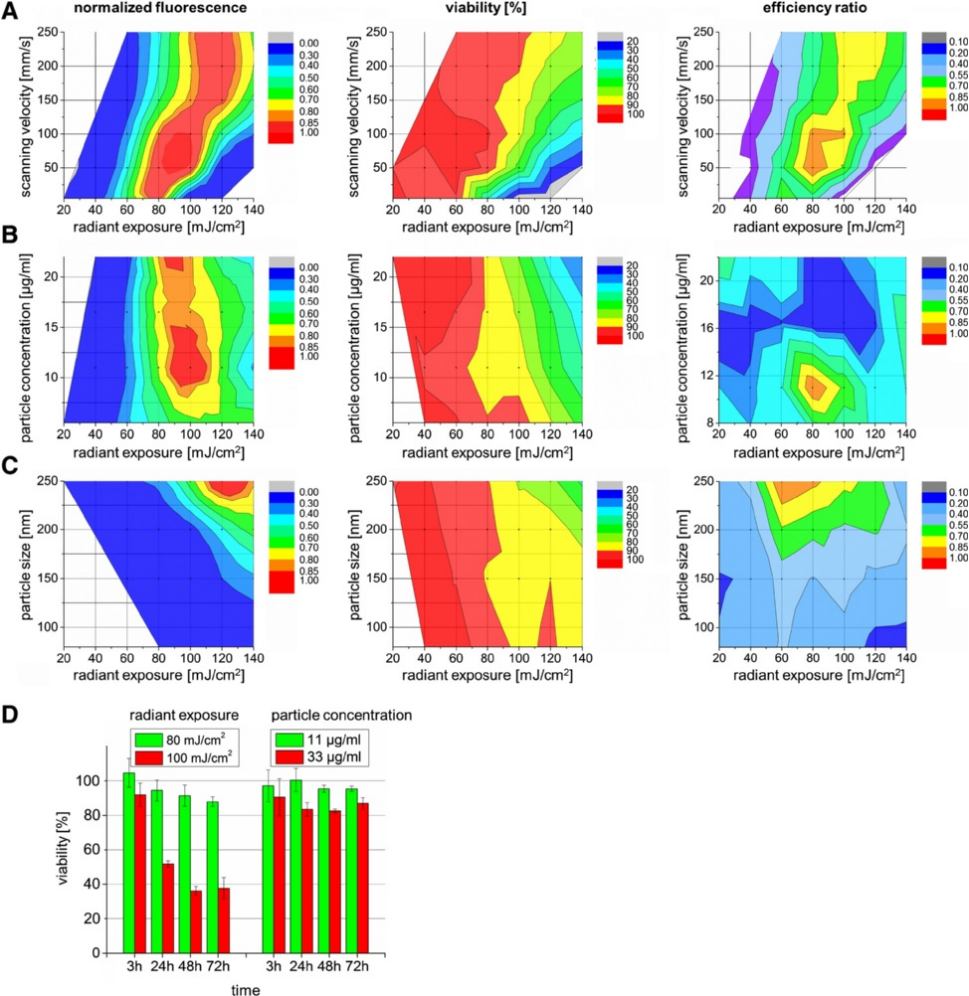
**Evaluation of efficient and gentle cell manipulation parameters.** Normalized fluorescence levels of delivered 10 kDa FITC dextran into ZMTH3 cells (left), the cell viability (middle) and the efficiency ratio (normalized ratio of FITC fluorescence level and viability) for different manipulation parameters in dependence of radiant exposure (20 mJ/cm^2^ to 140 mJ/cm^2^). **A)** Different scanning velocities at a defined AuNP concentration of 11 μg/ml and particle size of 250 nm, **B)** particle concentrations at a defined scanning velocity of 50 mm/s and particle size of 250 nm and **C)** different particle sizes (80 nm, 150 nm, 200 nm, 250 nm) at a defined AuNP concentration of 11 μg/ml and scanning velocity of 50 mm/s. **D)** Viability of cells incubated with AuNP for a time span up to 72 h after treatment with different radiant exposures (left) and different AuNP concentrations without laser exposure (right). The respective data points represents the mean value ± standard error of n = 4 independent experiments in triplicates on different days.

The influence of the scanning velocity on the molecular uptake targeting ZMTH3 cells is shown in Figure 3A. At a fixed scanning velocity, AuNP size and AuNP concentration of 11 μg/ml, the FITC fluorescence level increased with increasing radiant exposure. The highest efficiency ratio was found at 80 mJ/cm^2^ for a scanning velocity of 50 mm/s. With higher radiant exposures, the efficiency ratio decreased due to a loss in cell viability as a consequence of an irreversible damage of the cell membrane and/or the ablation of the cells from the glass bottom. Varying the AuNP concentration (Figure 3B), the highest efficiency ratio was reached at a concentration of 11 μg/ml (6.3 μg/cm^2^) and a radiant exposure of 80 mJ/cm^2^. When exceeding the threshold of 11 μg/ml the efficiency ratio dropped most likely due to many induced pores which consequently results in irreversible cell damage.

A further parameter impacting the efficiency ratio is the particle size (Figure 3C). A higher efficiency ratio was reached with an increase of particle size. Up to a particle size of 150 nm no efficient permeabilization occurred. Using larger particle sizes, the efficiency ratio peaked at a radiant exposure of 60 mJ/cm^2^ for 200 nm and 60–80 mJ/cm^2^ for 250 nm particles before the efficiency ratio dropped due to laser induced cell damage.

### Monitoring of the exposure effects on cell viability

The cell viability after performing permeabilization experiments at different radiant exposures and a fixed AuNP concentration of 11 μg/ml was followed up to 72 h. As presented in Figure 3D (left), the cell viability remained above 80% using radiant exposures up to 80 mJ/cm^2^. For a higher radiant exposure of 100 mJ/cm^2^ the cell viability strongly decreased to 40%.

The incubation of cells with AuNP at a concentration of 11 μg/ml for three hours without laser treatment did not show any pronounced effect on the viability for a time period of 72 h. Even the tripling of the AUNP incubation concentration to 33 μg/ml leads only to a slight decrease to 80-85% in cell viability. This negative effect on cell viability is likely to be caused by the residues of chloroauric acid used while particle manufacture.

Based on the presented results in Figure 3, the optimal parameter for an efficient cell permeabilization and tolerable cell loss is to a radiant exposure of 80 mJ/cm^2^, a particle size of 250 nm and an AuNP concentration of 11 μg/ml.

### Nanoparticle mediated laser transfection

**Figure 4:**
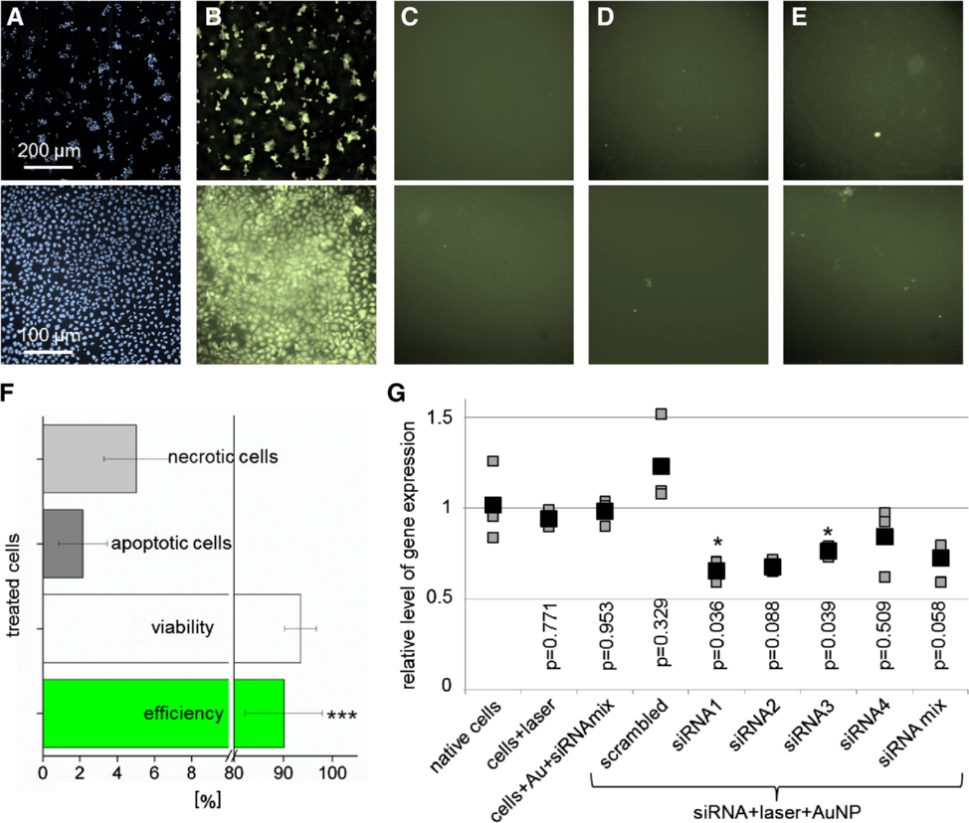
**siRNA transfection. A-E)** Microscopic images of treated cells (upper row CT1258 cells, bottom row ZMTH3 cells): **A)** The fluorescent images of Hoechst stained cell nuclei shows cell density. **B)** An Alexa fluor 488 labeled siRNA was efficiently transfected into the cells. **C)** The incubation of cells with siRNA (negative control) and **D)** the fluorescent image of AuNP labeled cells (AuNP control) show no siRNA uptake. **E)** A slight fluorescence signal in the laser control is detected when native cells are irradiated with the laser in the presence of the fluorescent siRNA. **F)** The flow cytometric analysis of siRNA transfected cells shows an efficiency of about 90% and a cell viability of 93%. Every data point represents the mean value ± standard error of n = 3 independent experiments in triplicates on different days. **G)** Real-time PCR analysis: Transfection of CT1258 cells results in HMGA2 gene knock down using different HMGA2 specific siRNAs (real-time PCR analyses were performed in triplicates). The black boxes represent the mean values of the qRT-PCR analysis and the grey boxes depict each of the three single measurements.

The evaluated parameters allowing an efficient and gentle cell permeabilization were used for cell transfection experiments. In Figure 4A the cell density is visualized by Hoechst 33342 nuclei staining. The successful transfection of CT1258 and ZMTH3 cells with an Alexa Fluor 488 labeled siRNA was performed using the optimized parameter (Figure 4B). Neither in the negative control (with siRNA, no laser treatment (Figure 4C)) nor in the AuNP control (with siRNA and AuNP incubation (Figure 4D)) a fluorescent signal was detected. Within the laser control (with siRNA and laser treatment, no AuNP) a weak fluorescence in individual cells was detected (Figure 4E). For CT1258 cells, a transfection efficiency of 85% ± 9 was evaluated using fluorescence microscopy. Here the fraction of necrotic cells was 3%. Flow cytometry analysis of ZMTH3 cells revealed a transfection efficiency of 90% and a cell viability of 93.5%. A significant difference (* p ≤ 0.05) was found between the siRNA samples and the native cells. The percentage of apoptotic cells was 2.15% and 5% for necrotic cells (Figure 4F).

In order to evaluate a potential gene therapeutic approach, functional siRNAs were used in a proof of principle experiment using high cell numbers. For HMGA2 (high mobility group AT-hook 2) gene knock down experiments the canine HMGA2 overexpressing cell line CT1258 [25] was transfected with four different anti-HMGA2 siRNAs complementary to the 3′-untranslated region of the HMGA2 mRNA and one non-sense scrambled siRNA. Due to the lack of reliable evaluated canine antibodies against the protein and thus potential unspecific cross reactions we opted for quantitative real-time PCR as detection method. This technique allows to measure the canine HMGA2 mRNA expression quantitatively.

The relative *HMGA2* mRNA expression was analyzed 48 h after treatment via one step quantitative real time PCR (qRT-PCR) analysis (Figure 4G). The *HMGA2* expression was quantified relative to the housekeeping gene *Beta-actin (ACTB)*, the non-treated cells were used for calibration (reference value = 1). In all samples treated with *HMGA2* specific siRNAs in combination with the laser manipulation suppression of *HMGA2* could be observed. The highest suppression was induced by using the siRNA 1 and 2. For the siRNA 1 and 3, the gene knock down was significant compared to native cells (p-values < 0.05). In the control samples, no *HMGA2* gene knock down could be observed. A slight increase was found for the scrambled siRNA, potentially resulting from off-target effects. No significant difference between the control samples and native cells was observed.

### Characterization of the nanoparticle mediated membrane permeabilization mechanism

In this section we describe different experiments to address the mechanisms involved in membrane permeabilization focusing on the parameters allowing an efficient and gentle cell manipulation.

**Figure 5:**
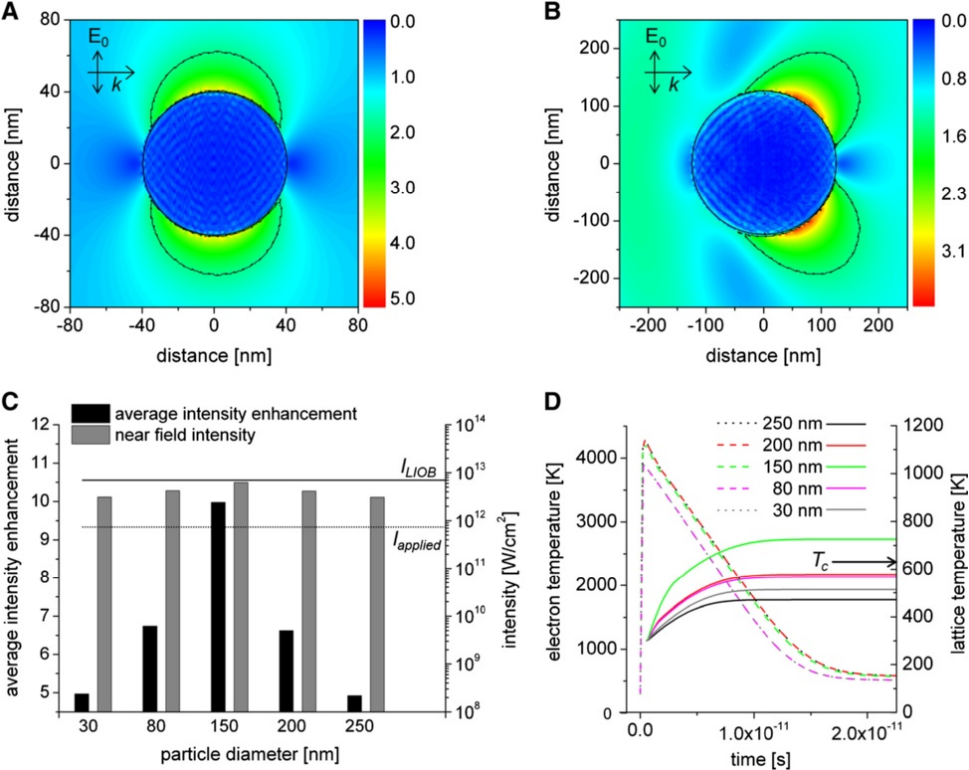
**Particle – laser interaction.** Particle excitation after interaction of a 798 nm laser pulse with duration of 120 fs and a radiant exposure of 80 mJ/cm^2^ (I = 6.26×10^11^ W/cm^2^). **A)** Map of the near field enhancement factor (η = E/E0) around an 80 nm particle and **B)** 250 nm particle. The dashed lines indicate the areas were the enhancement factor is higher than 1/e. The incident field Eo propagates along the x axis. **C)** Calculations of the near field enhancement around AuNP (left y axis) and the near field intensity (right y axis) for different AuNP sizes. Additionally the threshold for the applied intensity (dashed line) and the calculated intensity for LIOB using a NA 1.3 focused laser spot (solid line) is drawn. **D)** Evolution of the electron- (dashed lines) and lattice temperature (solid lines) after laser pulse absorption. The critical temperature Tc when water becomes hydro-dynamically unstable is indicated at 647 K.

Simulations of the near field distribution of the electric field at an incident wavelength of 796 nm for 80 nm and 250 nm particles are shown in Figure 5A and B, mapping the field-enhancement at the particles. For larger particles the dipole emission is distorted due to multipole oscillations within the sphere [17]. The enhancement factors of the different AuNP sizes are presented in Figure 5C (left y axis). For the used particles the highest field enhancement is reached for 150 nm particles. Here the near field is about 10 times higher than the incident field. For the 200 nm and 250 nm particles, the average enhancement is at 6.6 and 4.9, respectively. Furthermore, the near field volume is increasing with an increasing particle size (Table 1) and thereby the interaction zone of the near field with the membrane. This could be a reason for the increasing permeabilization efficiency at larger particle sizes shown in Figure 3C.

**Table 1:** **Near field and temperature related values for AuNP irradiated with 796 nm and 6.26 W/cm** ^**2**^

	**30 nm**	**80 nm**	**150 nm**	**200 nm**	**250 nm**
Near field volume [nm^3^] (I_max_/e^2^)	7×10^3^	1.2×10^5^	7.1×10^5^	1.6×10^6^	2.8×10^6^
Average near field intensity [W/cm^2^]	3.8×10^12^	2.5×10^12^	1.7×10^12^	2.5×10^12^	3.3×10^12^
Absorption efficiency Q_abs_	0.025	0.05	0.145	0.125	0.08
Particle temperature [K]	541	561	726	578	471

The evaluated enhancement factors and the applied intensity were used to calculate the near field intensity (Figure 5C (right y axis)). The values of the near field intensities of all particle sizes are below the threshold of an optical breakdown (LIOB) in water, which is 6×10^12^ W/cm^2^ for the used wavelength and pulse duration [26]. The highest near field intensity is reached for 150 nm particles which is close to the LIOB threshold. Intensities below the LIOB threshold in the low density plasma regime can lead to nonlinear effects like multiphoton ionization and avalanche-ionization. This might lead to the permeabilization of the cell membrane [14,22,27].

The accumulation of single pulses can induce the dissociation of biological molecules by forming reactive oxygen species (ROS) which results in membrane permeabilization [27]. Here, the threshold pulse energy E_N_ depends on the number of pulses (Equation (1)) [14,27].

1 $$ {E}_N={E}_1\cdot {N}^{-1/k}. $$

Where E_1_ describes the threshold energy of a single pulse, N is the number of pulses and k the accumulation strength [28].

**Figure 6:**
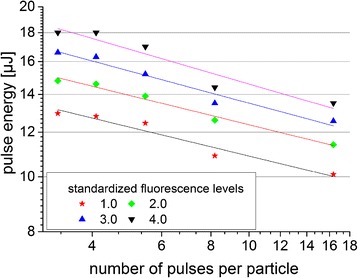
**Influence of the pulse energy on the number of pulses for standardized fluorescence levels.** Every data point represents the mean value (n = 15 measurements).

The dependence of the pulse energy on the number of pulses for standardized fluorescence levels (“fluorescence brightness”) is shown in Figure 6. A given fluorescence level corresponds to a specific amount of fluorescence molecules in the cells per well. We analyzed the number of laser pulses and pulse energy to yield four different fluorescence levels.

With an increasing number of pulses, less pulse energy is needed for an efficient permeabilization. An average accumulation strength of k = 5.57 ± 0.02 was evaluated by a power-law fit (Table 2). This indicates that 5 photons with a photon energy of 1.55 eV at the applied wavelength of 796 nm are absorbed simultaneously to reach the ionization threshold of 6.6 eV for water [26].

**Table 2:** **Power-law fit**

	**Threshold energy E**_**1**_ **[μJ]**	***k***	**R** ^**2**^
1.0	16.06 ± 0.7	5.88 ± 0.02	0.93
2.0	18.36 ± 0.7	5.88 ± 0.01	0.98
3.0	20.79 ± 0.7	5.55 ± 0.02	0.94
4.0	23.27 ± 0.7	5.00 ± 0.03	0.90

Dependence of the pulse energy on the number of pulses for standardized permeabilization efficiencies.

When laser radiation is absorbed by the electrons of the AuNP the energy is transferred from the electrons to the particle lattice due to electron phonon coupling within a time span of a few ps and the particle temperature increases [29,30]. The temperature of the electrons and the lattice can be calculated with a two-temperature model (Figure 5D) [31]. The lattice temperature reaches the highest temperature of 726 K for 150 nm particles. This temperature is above the critical temperature (T_c_) for phase transformation in water. For all other particles sizes this critical temperature is not reached. This reflects the different values for absorption efficiencies Q_abs_ listed in Table 1.

**Figure 7:**
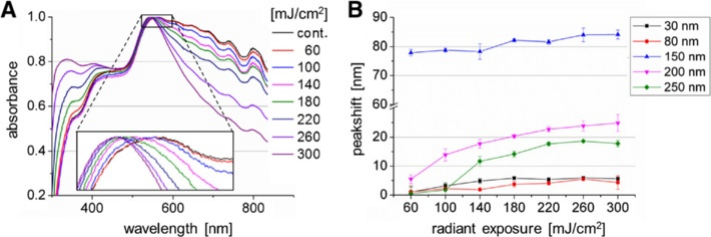
**Influence of different laser radiations on AuNP. A)** Absorbance spectrum of 250 nm particles after laser exposure. **B)** Mean value of the peak shift ± SEM in the absorbance spectrum after laser exposure for different AuNP sizes (n = 4 experiments).

The influence of the laser irradiation and possible changes in the particle morphology due to melting or fragmentation were analyzed by absorbance spectra of irradiated and non-irradiated particles (Figure 7A). After irradiation of 250 nm particles with radiant exposures of 60 mJ/cm^2^ and 100 mJ/cm^2^ a blue shift of the peak of 0.5 nm and 1.75 nm, occurred respectively (Table 3)). These shifts were in the SEM range of the untreated control. A reason for small changes can be polishing effects due to surface melting of the particle, which occurs below the melting point of bulk gold [17]. At radiant exposures of 140 mJ/cm^2^ and higher values the spectrum were broadly blue shifted (Table 3) and a narrowing of the spectrum occurred (Figure 7A). The spectrum of a 250 nm particle exposed to a radiant exposure of 300 mJ/cm^2^ is broadly similar to the spectrum of 80 nm particles [32]. This clearly indicates a change in particle size due to laser exposure which can be induced by particle evaporation or near field ablation [17].

**Table 3:** **Mean values ± SEM for the peak shift in the absorbance spectrum**

**Radiant exposure [mJ/cm** ^**2**^ **]**	**0**	**60**	**100**	**140**	**180**	**220**	**260**	**300**
Shift [nm]	0	0.5	1.75	11.68	14.18	17.68	18.62	17.81
SEM [nm]	±2.98	±2.68	±2.21	±1.71	±1.01	±0.66	±0.13	±1.06

Irradiation of 250 nm particles with different radiant exposures (n = 4 experiments).

The value of the peak shift for different particle sizes after laser radiation in dependence of the radiant exposure is shown in Figure 7B. The highest peak shift of 80 nm was measured for 150 nm particles and barley changes with increasing radiant exposures. Relating to the AuNP size of 250 nm used for transfection a peak shift occurred at radiant exposures ≥ 140 mJ/cm^2^. Furthermore, the amount of the peak shift for all radiant exposures is lower than for 150 nm particles which correlate with the calculated temperatures and near field enhancement in Figure 5.

## Discussion

In our study, we characterized the underlying mechanism and the potential of nanoparticle mediated cell membrane perforation in combination with fs-laser pulses as an alternative optical transfection method. Therefore the influencing parameters on the achieved perforation rate and cell viability were systematically determined and the successful transfection of cells with a fluorescent siRNA as well as the knock down of the oncogene *HMGA2* in tumor cells with specific siRNAs was demonstrated. Furthermore, the passive binding of AuNP to the cell membrane was studied.

Multiphoton and scanning electron microscopy images show the localization of AuNP near the cell membrane. Depending on the incubation time of the AuNP, single particles or clusters are located near, or associated with, the membrane. After an incubation time of 3 h the AuNP are clearly visible near the cell membrane. Within this time the particles form clusters with enhanced scattering of the laser light proved by multiphoton microscopy [33]. The agglomeration of particles after 3 hours is also visible in the ESEM images. This is in agreement with findings from Chithrani et al. who determined the uptake half-life at 2.24 h for 74 nm AuNP [33]. Furthermore, they evaluated the uptake of the number of particles per cell and also showed that the number of particles per cell saturated after 5 h. In the present study a particle number of approx. 160 was estimated at the membrane of a single cell for an incubation concentration of 11 μg/ml. Baumgart et al. [22] counted per cell 90 ± 23 AuNP with a diameter of 100 nm at an incubation concentration of 8 μg/ml after an incubation time of 6 h using SEM images. Taking into account that in this work a higher particle concentration and a larger diameter was used (and therefore a faster sedimentation of the particles takes place) the results are in a very good agreement.

In addition, Chithrani et al. evaluated the number of AuNP per vesicle and found an average number of 3 AuNP per vesicle for 100 nm particles. In comparison to our SEM images (see Figure 2B) we assume that one 200 nm particle per vesicle get endocytosed by the cell. As bare AuNP are used, a serum protein corona is formed at the particles surface and no specific binding at the cell membrane is likely to occur. Therefore, we suggested the receptor-mediated endocytosis (RME) to be the acting uptake mechanism [34].

The initial mechanism of plasmon mediated cell membrane permeabilization is still a current matter in research. Depending on the parameters, different mechanisms and effects are assumed. These are thermal (“nanoheater effect”) [19,35], or near field enhancement effects (“nanolens effect”) [18,35]. In addition, the generation of a low density plasma induced by multiphoton ionization combined with thermal effects can possibly lead to membrane permeabilization [14,15]. For short laser pulses in the nanosecond-picosecond regime, where the energy is mainly absorbed by the particle, thermal effects could be the main mechanism for membrane permeabilization [36-38]. After AuNP heating, the water evaporates followed by a shockwave and forming a cavitation bubble around the exposed particles as reported by Pitsillides et al. and Zharov et al. and enabling membrane perforation [39,40]. Using fs laser pulses, nanocavitation bubbles can be formed by the induced field enhancement. This enhancement can lead to an optical breakdown near the particle and to the generation of a shockwave [18,36]. In this work, the evaluated intensities at the surface of single AuNP are near the threshold for an optical breakdown in the low density plasma regime. In existing studies, different concentrations of AuNP were required to achieve cell membrane perforation [15]. Higher numbers of particles are necessary to manipulate the cells with fs laser pulses [22,41]. Due to the formation of AuNP clusters the near field is further enhanced in comparison to single particles. The neighboring particles interact via the scattered waves and due to plasmon coupling “hot spots” are formed [42,43]. Taking into account that the field enhancement is higher for AuNP clusters compared to single particles, the intensities could be above the optical breakdown threshold [42].

Our assumption herein, that clusters of AuNP at the cell membrane are necessary to induce a field enhancement by fs laser pulses which is high enough to perforate the cell membrane. This is supported by the presented microscopic images and the number of AuNP utilized in this and other studies using fs laser pulses for membrane perforation [22,41]. Within the performed experiments we showed the efficient and transient permeabilization of the cell membrane due to an expected enhancement of the near field at the AuNP clusters. Based on this and the evaluated simultaneous absorption of 5 photons in the pulse number dependent experiments (Figure 6) we understand the near field enhancement followed by the multiphoton ionization of the surrounding medium as the initial perforation mechanism.

**Figure 8:**
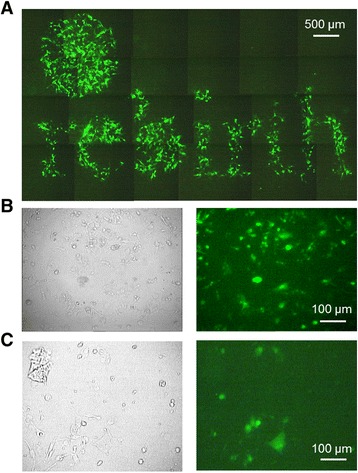
**Spatial selective and pluripotent cell line manipulation. A)** Selective cell manipulation of ZMTH3 cells by spatial confined radiation using a shadow mask. The image consists of 24 single fluorescence images and shows the word “rebirth” **B-C)** Manipulation of a human ES cell line hES3 **(B)** and the human iPSC line hCBiPS2 **(C)**: A membrane impermeable dye (Lucifer yellow) was delivered into the pluripotent cells. Bright field image (left) and fluorescence image (right).

The fs laser pulses are enhanced in the near-field of the particle for membrane permeabilization by surface plasmon resonances. NIR fs laser pulses benefit from a low thermal impact and a high penetration depth into tissue which is important for *in vivo* experiments. Furthermore, laser irradiation mediated fragmentation of nanoparticles is especially for *in vivo* settings an important issue. Fragmentation in small nanoparticles under 5 nm can lead to toxicity by intercalation into the DNA [44]. The comparison of fs pulses and ns pulses reveal a more pronounced change in particle morphology for longer (850 ps, 532 nm) pulses. In absorbance measurements, no pronounced peak shift (as indicator for particle morphology and size change) was detected for a fluence of 100 mJ/cm2, which exceeds the optimal fluence for cell manipulation (80 mJ/cm2) using NIR fs pulses (Table 3). In comparison to this, for 850 ps (≈1 ns) pulses a peak shift of 15.4 nm was determined using the optimal manipulation fluence of 20 mJ/cm2. The change in particle morphology was caused due to thermal effects and the strong linear absorption at 532 nm. Nevertheless, the cell viability stayed above 80% in all the performed experiments suggesting the use of visible ns pulses for *in vitro* experiments [15]. For fs pulses, the cell viability was determined to be above 90%. The presented results and the advantages of NIR fs laser pulses (e.g. a high penetration depth and the avoidance of photo thermal effects) indicate the great potential of fs laser for *in vivo* manipulation. Furthermore the development of endoscopic systems for ultrafast laser microsurgery [45] or fiber based approaches [46] makes the application of ultrashort laser pulses potentially suitable for fs laser *in vivo* cell manipulation. Additionally, first *in vivo* experiments showed the generation of nanobubbles around AuNP clusters for selective cancer cell killing using short, 780 nm laser pulses [47]. Next to the properties of the fs laser pulses, an advantage of the presented method is the double selectivity by the spatial confined radiation (Figure 8A) and the possibility of specific cell targeting by antibody conjugated AuNPs. The latter can be used to induce selective cell manipulation or ablation in both, *in vitro* and *in vivo* models. For example, the treatment of squamous carcinoma cells in the buccal mucosa or at the tongue. Further in tumor scenarios where minimal invasive tissue ablation is essential as malignant glioblastoma, it is crucial to sustain non-target (healthy) tissue. Here the presented method can be a powerful tool. Exemplarily for primary cell manipulation a membrane impermeable fluorescent dye was delivered into a human embryonic stem cell (ES) cell line hES3 and a human induced pluripotent stem cell (iPSC) line hCBiPS2 (Figure 8B).” The results of the siRNA experiments show that fs nanoparticle mediated laser transfection is suitable for high throughput functional gene assays due to the short processing time of approximately 10 min per 96 well plate. As the applied AuNP were shown to be non-toxic, this method is excellent suited for *in vitro* application but also for other applications in molecular medicine. Furthermore, it can be applied for the manipulation of various cell types as shown in our previous work and by Baumgart et al. [16,22]. Additional applications as gene or cell therapeutic approaches can be served by this technique. As an example, it is possible to manipulate high cell numbers required for e.g. tumor vaccination strategies in an appropriate time. The knock down of the oncogene HMGA2 in canine prostate carcinoma cells was carried out successfully as shown by real time PCR expression analyses (Figure 4G). Due to the extraordinary high HMGA2 expression in the CT1258 cell line a incomplete siRNA mediated HMGA2 knock down within the treated cells was to be expected. Conventional HMBA2 knock down in less aggressive human pancreatic cell lines by Watanabe et al. [48] resulted in higher efficiencies. However, we opted to target the canine prostate cancer derived cell line CT1258 as canine prostate cancer represents the only spontaneously arising model for human prostate cancer with considerable incidence. This includes several tumor relevant aspects as biological behavior, marker gene expression and histological presentation [49]. Thus, a successful establishment of a therapeutic approach in dogs will offers high transfer potential to a human clinical setting. Consequently, prior to human clinical trials, a valid clinical trial in dogs as naturally occurring model is of major interest allowing to monitor the therapeutic intervention in an genetic outbreed model with unmanipulated immune system.

## Conclusion

Our studies on nanoparticle mediated fs laser cell perforation show, that this method is suitable for high throughput siRNA transfection with high efficiency and low cell toxicity. To establish this method as an alternative transfection technique, the manipulation of different cell types will be continued in further studies. However, due to the underlying physical mechanism the permeabilization should be cell type independent. Based on the mechanistic investigations, we assume that an enhancement of the near field occurs at AuNP clusters. This leads to the generation of a low density plasma with multiphoton ionization of the surrounding liquid, which in turn perforates the cell membrane. The uptake mechanism of extracellular molecules remains to be investigated in further experiments [50]. The presented method is an alternative transfection method to deliver molecules into living cells being particularly well suited for standardized processes like high throughput or high content screening assays for fundamental and pharmaceutical research.

## Methods

### Cell culture

The canine pleomorphic mammary adenoma cell line ZMTH3 [51] was cultured routinely in RPMI 1640 supplemented with 10% fetal calf serum (FCS) and 1% penicillin/streptomycin (Biochrom AG, Berlin, Germany). Rat granulosa cells (GFSHR-17) were cultivated in DMEM (Dulbecco’s Modified Eagle Medium) supplemented with 5% FCS, 1% penicillin/streptomycin (Biochrom AG, Berlin, Germany). The canine prostate adenocarcinoma cell line CT1258 was derived from an extremely aggressive canine prostate carcinoma [52] and cultured in Medium 199 (Life Technologies GmbH, Darmstadt, Germany) supplemented with 10% FCS and 2% penicillin/streptomycin (Biochrom AG, Berlin, Germany). The human ES cell line hES3 and the human iPSC line hCBiPS2 [53] were cultured and expanded on irradiated mouse embryonic fibroblasts (MEF) in knockout DMEM supplemented with 20% knockout serum replacement, 1mM L-glutamine, 0.1mM β-mercaptoethanol, 1% nonessential amino acid stock (all from Life Technologies) and 10ng/ml bFGF (supplied by the Institute for Technical Chemistry, Leibniz University Hannover). One day before laser transfection cells were detached from the feeder layer by 0.2% collagenase IV (Life Technologies) followed by an incubation step with TrypLE (Life Technologies) for single-cell dissociation and plated onto Matrigel™ (BD Biosciences) coated dishes in MEF-conditioned medium.

### Laser setup

The used automated setup for cell manipulation is operating with a fs amplifier laser system (Spitfire Pro, Newport Corporation, Irvine, USA). The generated laser pulses have a pulse duration of 120 fs at a fixed wavelength of 796 nm. The output power of the system is 2.1 W at a repetition rate of 5 kHz. To irradiate the biological tissue, the laser pulses were guided through an automatized attenuator consisting of a λ/2-plate and a polarizing beam splitter and reflected by two scanning mirrors (Litrack, JMLaser, Müller Elektronik, Spaiching, Germany). A convex lens with a focal length of 800 mm was used to focus the laser pulses onto the sample, located on the automatized stage (OptiScan, Prior, Jena, Germany), resulting in a spot diameter of 164 μm.

### Nanoparticle incubation

Prior to the laser cell manipulation experiments and to investigate the interaction of AuNP with the cell membrane, the cells were co-incubated with the AuNP at 37°C in a 5% CO_2_ atmosphere. The AuNP were chemically manufactured in presence of chloroauric acid (PGO, Kisker Biotech, Steinfurt, Germany). Uncoated AuNP of 80 nm, 150 nm, 200 nm, 250 nm were used.

### Multiphoton microscopy

Images were obtained to evaluate the incubation time for AuNP mediated cell permeabilization and the possibility of a passive binding of the particles. Briefly, granulosa cells were incubated with 150 nm gold particles and imaged after different incubation times. After a PBS wash, the cells were observed with a custom built multiphoton microscope which is based on a fs-laser system tunable from λ = 690 nm to 1040 nm (Chameleon ultra II, Coherent, Göttingen, Germany) [27]. The images were recorded through a 100× oil immersion objective (Carl Zeiss AG, “Plan-Neofluar”, NA = 1.3) at an excitation wavelength of λ_exc_ = 700 nm.

### Scanning electron microscopy (SEM) and environmental scanning electron microscopy (ESEM)

To investigate the interaction of cells and AuNP images of ZMTH3 cells were generated after different times of co-incubation with 200 nm particles. The cells were washed after co-incubation with AuNP and fixed by adding a 4% paraformaldehyde (PFA) solution with 0.2% glutaraldehyde at 4°C. For ESEM imaging the cells were washed after 20 min with distilled water. For SEM, the cells were further treated at room temperature for 20 min with a 2% osmium tetroxide solution. Subsequently, the cells were washed 3 times with water for 5 min before incubation with different ethanol concentrations for 10 min each (30%, 50%, 70%, 90%, 95%, 95% and 3 × 100%). Before sputtering the cells with a 5 nm gold layer, the cells were dried for 30 min under laminar air flow conditions. For counting AuNP at the cell membrane after incubation, ImageJ was used [54]. Values represent the mean of n = 6 images ± SEM.

### Plate reader measurements

To evaluate the optimal parameters of an efficient and gentle transfection, 2.5×10^4^ canine ZMTH3 cells per well were seeded in a black wall/clear bottom 96 well plate (BD Bioscience, Heidelberg, Germany) 24 h before laser treatment. As an indicator of membrane permeabilization, fresh medium with 2 mg/ml of 10 kDa FITC-dextran (Sigma-Aldrich, Steinheim, Germany) was added to the cells. After laser treatment, the cells were incubated for 30 min followed by several washing steps until the background fluorescence from the permeabilization indicator (10 kDa FITC dextran) was eliminated. To measure the metabolic activity of the cells, 10% (v/v) of the resazurin based, fluorometric QBlue viability assay kit (BioCat GmbH, Heidelberg, Germany) was added to the medium. During an incubation time of 1 h, viable cells converted resazurin into the fluorescent form resuorufin. The fluorescence levels of the delivered FITC dextran (EX488/EM520 nm) for molecular delivery and the resorufin (EX570/EM600 nm) as an indicator for viability were measured by the Infinite 200 Pro plate reader (Tecan, Männedorf, Switzerland). The value for FITC dextran delivery was calculated by subtracting the fluorescent background from each sample and afterwards the highest FITC fluorescence level was normalized to 1. The cell viability (V) was determined by the QBlue fluorescence level of the sample (F_s_), the fluorescence of the untreated control (F_C_) and the background (F_B_) (Equation(2)).

2 $$ V=\frac{F_S-{F}_B}{F_C-{F}_B}\cdot 100 $$

The efficiency ratio (E) was calculated by correlating the fluorescence level for molecular delivery (F_FITC_) and viability (V) using equation (3). Afterwards the values were normalized to 1.

3 $$ E={F}_{FITC}\cdot V $$

### Simulation of the particle temperature and near field

For a deeper insight into the mechanisms involved in membrane permeabilization using fs laser pulses, the particle temperature and the near field were analyzed. The temperature of the AuNP during fs irradiation was calculated based on a two temperature model, employing data for the specific heat capacity of the electrons and the electron phonon coupling constant from Lin et al. [55]. Temperature loss due to interaction with the surrounding medium was not considered due to the short timescales used. The field strength and intensity as well as the near field volume were simulated by the discrete dipole approximation, using the software DDSCAT [56,57]. A dipole separation of less than 3.5 nm was used for the largest sphere with a diameter of 250 nm. Modeling of the optical breakdown intensities in the near field was performed according to the Keldysh theory following the approach used by Vogel et al. [26,58]. The maximum intensity divided by the square of e was considered as near field volume and the enhancement in the modeling of the optical breakdown as well as the near field volume were averaged in the according area.

### UV–Vis spectroscopy

Particle spectra were monitored to evaluate a possible peakshift (as an indicator for a change in particle size/shape) of laser irradiated particles compare to untreated particles. Therefore an UV/Vis spectroscope (UV 1650-PC, Shimadzu, Duisburg, Germany) was used. The particles were diluted in culture media (RPMI as described before) without phenol red at a concentration of 50 μg/ml. Using a 96 well plate, the samples with a total volume of 200 μl per well were irradiated in a meander pattern.

### Fluorescence microscopy

In order to evaluate the transfection efficiency of the CT1258 cells, fluorescence microscopy was applied. 24 h before laser treatment, 1×10^4^ cells were seeded in each well of a 24 well plate (PAA Laboratories, Cölbe, Germany). For siRNA transfection, 10 μM of a fluorescently labeled (AlexaFluor488) siRNA (Qiagen, Hilden, Germany) was added to the extracellular medium before laser treatment. The samples were treated with the optimized parameters as evaluated within the plate reader measurements. After laser treatment, the cells were incubated for 30 min followed by several washing steps until the background fluorescence from the fluorescent siRNA was eliminated. Three independent experiments in duplicates were performed on different days. Three images of each well were analyzed using Image J. By counting the cell nuclei (ca. 546 per image, stained with HOECHST33342) and transfected cells (Alexafluor488 siRNA positive cells) the transfection efficiency was determined. Propidium Iodide was used as an indicator for necrotic cells.

### Flow cytometry analysis

Flow cytometric analysis was performed to evaluate the transfection efficiencies and the necrotic- and apoptotic rate. 24 h before laser treatment, 1.5×10^5^ cells were seeded in each well of a 24 well plate. For siRNA transfection, 10 μM of a fluorescently labeled (AlexaFluor488) siRNA was added to the extracellular medium before laser treatment. The samples were treated with optimized parameters as evaluated within the plate reader measurements. Three hours after laser treatment the samples were prepared for flow cytometric analysis. Therefore, the cells were washed and trypsinized (TrypLE™, Life Technologies (LT), Darmstadt, Germany). A viability staining with Annexin V (V-PE-Cy5 Apoptosis Detection Kit, BioCat, Heidelberg, Germany) to detect the apoptotic cells, and with 1.5 μM Propidium Iodide (Invitrogen, Darmstadt, Germany) to identify necrotic cells, was performed. The positivity of siRNA transfected cells was determined by comparing the AlexaFluor488 fluorescence intensity to native cells, both measured in the FL1-H channel using a FACSCalibur flow cytometer (BD Bioscience, Heidelberg, Germany). Within the native cell population, a gate was set determining 98% of the native cells as non-transfected using the software Cell Quest (BD Bioscience, Heidelberg, Germany). This gate was subsequently applied on the siRNA transfected cell population resulting in the percentage of positive and non-transfected cells. To determine the ratio of apoptotic and necrotic cells within the siRNA transfected samples, the Annexin V and PI labelled cells were analyzed for PE-Cy5 fluorescence in the FL4-H channel and for PI in the FL2-H channel. Within the native cells a gate was set at which a cell population of 2% was identified as Annexin and PI positive and transferred to the sample with siRNA transfected cells to discriminate living from apoptotic and necrotic cells. For statistical analyses, the student’s t-test was used. The significance is given as * for p < 0.05, ** for p < 0.01 and *** for p < 0.001.

### HMGA2 suppression analysis

As a proof of principle, that the presented method is suitable for molecular medicine approaches a functional gene knock down experiment was performed. We used the tumor cell line CT1258 which is characterized by overexpression of endogenous HMGA2 [25]. 24 h prior to transfection 3×10^5^ cells were seeded per well into a 6 well plate (Greiner Bio-One GmbH, Frickenhausen, Germany). Cells were laser-transfected with 10 nM of different anti-HMGA2 siRNAs, a scrambled siRNA and a siRNA mix consisting of 10 nM of each of the four anti-HMGA2 siRNAs (Riboxx, Radebeul, Germany). The corresponding siRNA sequences are listed in Table 4. After a time span of 48 h the growth medium was removed from the CT1258 cells and 1 ml Tryp LE Express (Life Technologies GmbH, Darmstedt, Germany) was applied on cells. Once the cells were detached 1 ml cultivation medium was added to stop the reaction. Cell suspension was pelleted at 300 × g for 5 min. The supernatant was discarded and the pellet stored at −80°C until further processing.

**Table 4:** **Name and sequence of the used siRNAs for HMGA2 knock down**

**siRNA name**	**sequence**
A2-3UTR 1	5′-UUAAUUCUCUCCGUAGCUCCCCC-3′
A2-3UTR 2	5′-UCUUACUGUUCCAUUGGCCCCC-3′
A2-3UTR 3	5′-AUUAUCCUUAAGAACCUAGCCCCC-3′
A2-3UTR 4	5′-UUCUUACUGUUCCAUUGGCCCCC-3′
scrambled siRNA	5′-UAAGCACGAAGCUCAGAGUCCCCC-3′

### RNA extraction

For PCR analysis total RNA was isolated according to the “NucleoSpin miRNA” protocol (Macherey & Nagel, Düren, Germany). Small and large RNAs were finally eluted in 30 μl nuclease free water. Total RNA concentration was measured with the Synergy 2 reader (BioTek Instruments GmbH, Bad Friedrichshall, Germany).

### Quantitative one step real-time PCR analysis

For the relative *HMGA2* / *ACTB* quantification 25 ng total RNA were mixed with SYBR Green, *HMGA2* or *ACTB* specific primers, nuclease free water (Qiagen, Hilden, Germany) and reverse transcriptase according to the “QuantiTect SYBR Green RT-PCR” protocol (Qiagen, Hilden, Germany). The fluorescence of each sample was analyzed in triplicates. As negative controls a non-template and a no-reverse transcriptase control were included. The experiments were performed using the Mastercycler ep realplex (Eppendorf AG, Hamburg, Germany). qRT-PCR conditions were as follows: 30 min at 50°C and 15 s at 95°C, followed by 40 cycles with 15 s at 94°C, 30s at 60°C and 30 s at 72°C. Finally a melting curve analysis was performed to verify specificity and identity of the qRT-PCR products according to the Eppendorf Mastercycler ep realplex instrument instructions. For the comparison of the relative gene expression levels based on the ∆∆CT method the gene expression level of the untreated CT1258 cells was used as calibrator (calibrator expression level was set as 1). Statistical analysis of the qRT-PCR results was done by using the software tool REST 2009, version 2.0.13. A p-value of ≤ 0.05 was considered as statistically significant.
